# Di­aqua­bis­(cinnamato-κ^2^ *O*,*O*′)cadmium

**DOI:** 10.1107/S160053681400364X

**Published:** 2014-02-26

**Authors:** Sirinart Chooset, Bryan Cunningham, Anob Kantacha, Matthias Zeller, Sumpun Wongnawa

**Affiliations:** a Department of Chemistry, Faculty of Science, Prince of Songkla University, Hat Yai, Songkhla 90112, Thailand; b Department of Chemistry, Youngstown State University, One University Plaza, Youngstown, OH 44555, USA; c Department of Chemistry, Faculty of Science, Thaksin University, (Patthalung Campus), Patthalung 93110, Thailand

## Abstract

The title complex, [Cd(C_9_H_7_O_2_)_2_(H_2_O)_2_], was obtained as an unintended product of the reaction of cadmium nitrate with hexa­methyl­ene­tetra­mine and cinnamic acid. The Cd^II^ ion lies on a twofold rotation axis and is coordinated in a slightly distorted trigonal–prismatic environment. In the crystal, the V-shaped mol­ecules are arranged in an inter­locking fashion along [010] and O—H⋯O hydrogen bonds link the mol­ecules, forming a two-dimensional network parallel to (001).

## Related literature   

For a previous conference report of the title compound, see: Amma *et al.* (1983 ▶). For related structures, see: Hosomi *et al.* (2000 ▶); Mak *et al.* (1985 ▶); Smith *et al.* (1981 ▶); O’Reilly *et al.* (1984 ▶). For a description of the Cambridge Structural Database, see: Allen (2002 ▶).
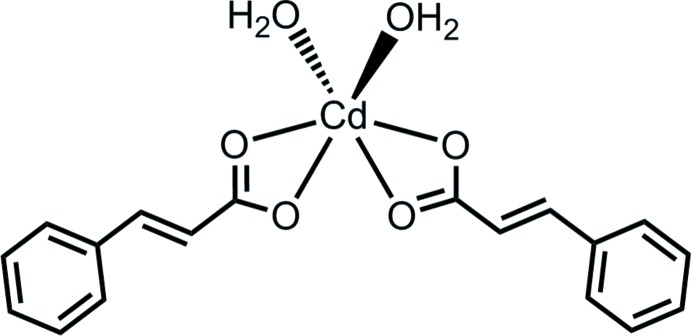


## Experimental   

### 

#### Crystal data  

[Cd(C_9_H_7_O_2_)_2_(H_2_O)_2_]
*M* *_r_* = 442.72
Monoclinic, 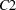

*a* = 11.7872 (12) Å
*b* = 5.3498 (5) Å
*c* = 13.8817 (14) Å
β = 99.913 (1)°
*V* = 862.30 (15) Å^3^
*Z* = 2
Mo *K*α radiation
μ = 1.30 mm^−1^
*T* = 100 K
0.28 × 0.09 × 0.02 mm

#### Data collection  

Bruker SMART APEX CCD diffractometer
Absorption correction: multi-scan (*SADABS*; Bruker, 2012 ▶) *T*_min_ = 0.617, *T*_max_ = 0.746
5087 measured reflections
2531 independent reflections
2529 reflections with *I* > 2σ(*I*)
*R*_int_ = 0.021

#### Refinement  

*R*[*F* ^2^ > 2σ(*F* ^2^)] = 0.020
*wR*(*F* ^2^) = 0.046
*S* = 1.05
2531 reflections
150 parameters
3 restraints
All H-atom parameters refined
Δρ_max_ = 1.09 e Å^−3^
Δρ_min_ = −0.42 e Å^−3^
Absolute structure: Flack parameter determined using 1059 quotients [(*I* ^+^)−(*I* ^−^)]/[(*I* ^+^)+(*I* ^−^)] (Parsons *et al.*, 2013 ▶)
Absolute structure parameter: 0.018 (14)

### 

Data collection: *APEX2* (Bruker, 2012 ▶); cell refinement: *SAINT* (Bruker, 2012 ▶); data reduction: *SAINT* and *SHELXTL* (Sheldrick, 2008 ▶); program(s) used to solve structure: *SHELXS97* (Sheldrick, 2008 ▶); program(s) used to refine structure: *SHELXL2013* (Sheldrick, 2008) ▶ and *SHELXLE* (Hübschle *et al.*, 2011 ▶); molecular graphics: *Mercury* (Macrae *et al.*, 2008 ▶) and *PLATON* (Spek, 2009 ▶); software used to prepare material for publication: *publCIF* (Westrip, 2010 ▶).

## Supplementary Material

Crystal structure: contains datablock(s) I, global. DOI: 10.1107/S160053681400364X/lh5688sup1.cif

Structure factors: contains datablock(s) I. DOI: 10.1107/S160053681400364X/lh5688Isup2.hkl

CCDC reference: 987495

Additional supporting information:  crystallographic information; 3D view; checkCIF report

## Figures and Tables

**Table 1:** Hydrogen-bond geometry (Å, °)

*D*—H⋯*A*	*D*—H	H⋯*A*	*D*⋯*A*	*D*—H⋯*A*
O3—H3*A*⋯O1^i^	0.82 (2)	1.86 (2)	2.679 (3)	174 (4)
O3—H3*B*⋯O2^ii^	0.80 (2)	1.86 (3)	2.658 (3)	171 (5)

Symmetry codes: (i) 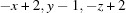; (ii) 
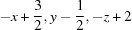
.

**Fig. 1.**
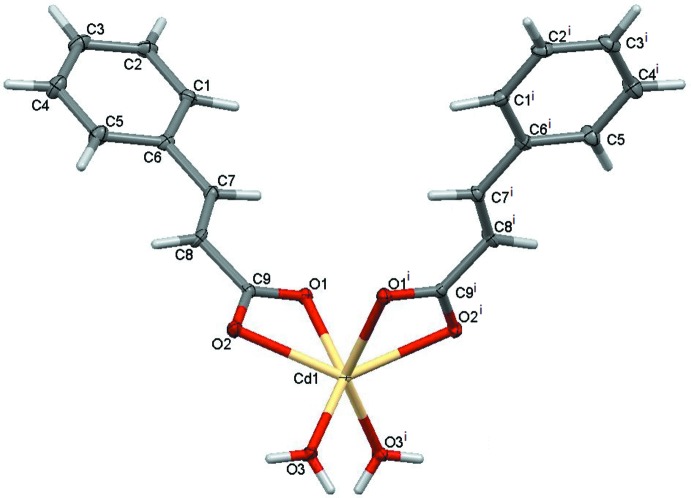
The molecular structure of the title compund, shown with ellipsoids at the 50% probability level. Symmetry operator (i): –x + 2, y, –z + 2.

**Fig. 2.**
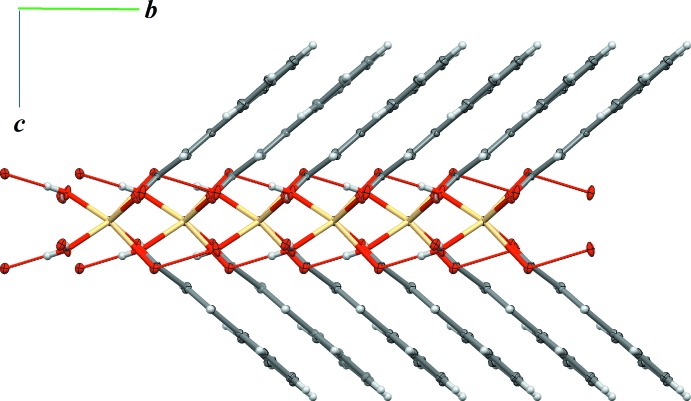
Part of the crystal structure showing molecules arranged along [010]. Hydrogen bonds are shown as blue dotted lines.

**Fig. 3.**
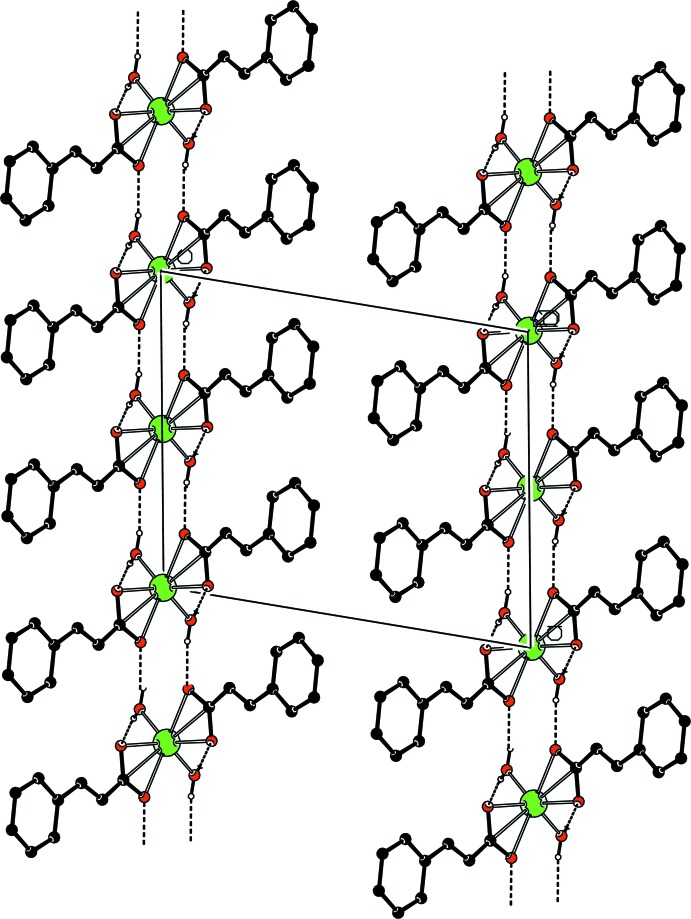
Part of the crystal structure showing layers perpendicular to the c-axis direction of the structure. Hydrogen bonds are illustrated by blue dotted lines.

**Fig. 4.**
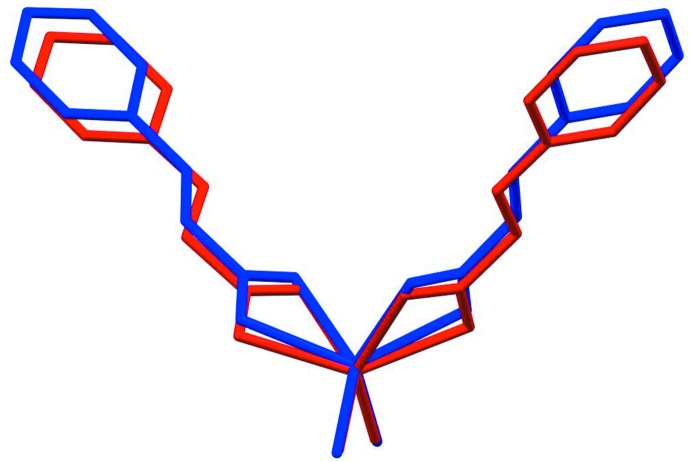
Overlaid stick presentation of diaqua-bis(cinnamate)-cadmium(II) (blue) and diaqua-bis(phenoxyacetato)-zinc(II) (red) (Smith et al., 1981).

**Fig. 5.**
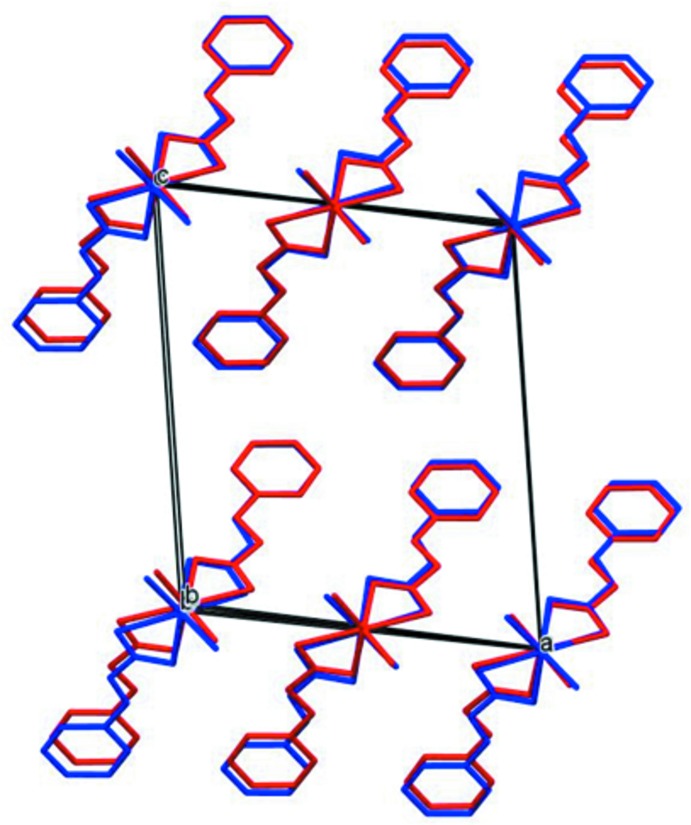
Overlaid stick presentation of diaqua-bis(cinnamic)-cadmium(ii) (blue) and diaqua-bis(phenoxyacetato)-zinc(II) (red) (Smith et al., 1981).

## supplementary crystallographic information

### 1. Comment

The title compound was obtained as an accidental product of the reaction of cadmium nitrate with hexamethylenetetramine and cinnamic acid in ethanol in an attempt to synthesize a potentially interesting framework compound of the metal with both tetraamine and carboxylic acid groups. The potentially bridging hexamethylenetetramine ligand may have acted as a linker between cadmium ions; however, it was not incorporated into the material. A mononuclear cadmium complex with water and cinnamate ligands was the product formed in 75% yield, from an ethanolic solution.

The structure of diaqua-bis(cinnamato)-cadmium(II) had been previously recorded and was presented at the 1983 meeting of the American Chemical Society, but complete structural details are not available (Amma *et al.*, 1983). In the Cambridge Structural Database (Version 5.35, with updates up to May 2013; Allen, 2002) [REFCODE: BUYTUK] only the data collection temperature (room temperature), unit cell parameters and space group, and the *R* value (10.4%) are reported but no atomic coordinates are available. Given the relatively poor precision of the previously reported structure and the lack of three-dimensional coordinates, we herein report the crystal structure of the title compound at 100 K.

The Cd^II^ lies on a two-fold rotation axis and is coordinated by two cinnamate ligands and two water molecules (Fig. 1). The carboxylate groups are bidentate-chelating, the water molecules monodentate and non bridging. The two oxygen atoms of each carboxylate group take coordination sites, the overall coordination environment of the metal center is best described as distorted trigonal prism, with angles varying between 92.86 (11)° (between the O atoms of the two water molecules), and 116.30 (8)° (for the angle between a water molecule O atom and a neighboring carboxylate group, using the carboxylate carbon atom as a substitute for the average of the two oxygen atoms).

The Cd—O bond distances are in the expected ranges. The bonds involving the water O atoms are 2.208 (2) Å , which compares well with those in similar Cd(II) complexes (O'Reilly *et al.*, 1984, Mak *et al.*, 1985). The Cd—O bond distances involving the two carboxylate O atoms are longer than those involving the water molecules, as would be expected due to the chelating coordination mode of the cinnamate ligand. The actual bond distances are 2.330 (2) and 2.375 (2) Å for Cd—O1 and Cd—O2, respectively. The similarity of the two Cd—O distances indicates an essentially symmetric coordination and a delocalization of the negative charge of the cinnamate carboxylate group. This is confirmed by the C—O bond distances within the carboxylate groups, which are also the same within experimental error, with values of 1.276 (3) and 1.269 (3) Å for O1—C9 and O2—C9, respectively.

In the crystal, the V-shape of the molecule results in a linear arrangement along [010] with the Cd(OH_2_)_2_ part of one molecule oriented towards the V-shaped part of a symmetry related molecule (Fig. 2). In addition, intermolecular O—H···O hydrogen bonds connect molecules forming a two-dimensional network parallel to (001) (Fig. 3).

A search against the Cambridge Structural Database provided several similar reported structures that are related to the title compound: the zinc derivative diaqua-bis(cinnamato)-zinc(II) (Hosomi *et al.*, 2000; CSD refcode KIYSEQ), and several of the zinc and cadmium phenoxyacetato derivatives: diaqua-bis(phenoxyacetato)-cadmium(II) (Mak *et al.*, 1985, csd refcode DEBGAS) and diaqua-bis(phenoxyacetato)-zinc(II) (Smith *et al.*, 1981, CSD refcode PHXCUB), diaqua-bis(4-fluorophenoxyacetato)-cadmium(II) (O'Reilly *et al.*, 1984; CSD refcode CUPMUV). Figures 4 and 5 show representative overlays of the title compound diaqua-bis(cinnamic)-cadmium(II) with diaqua-bis(phenoxyacetato)-zinc(II) (Smith *et al.*, 1981), indicating the isomorphous nature of the two compounds.

### 2. Experimental

To a stirred colorless solution of Cd(NO_3_)_2_·4H_2_O (0.3084 g, 1 mmol) in 10 mL of water was added hexamethylenetetramine (0.2802 g, 2 mmol) in 5 mL of water to give a colorless solution. Then, cinnamic acid (0.2962 g, 2 mmol) in 20 mL of ethanol was added to a give a colorless solution. The solution was stirred at room temperature for 6 h, was filtered and then left to evaporate at room temperature. After several days, colorless needle shaped crystals suitable for X-ray analysis were obtained in 75% yield. A single-crystal was isolated while suspended in mineral oil, was mounted with the help of a trace of mineral oil on a Mitegen micromesh mount and flash frozen to 100 K on the diffractometer.

### 3. Refinement

Reflection 0 0 1 was affected by the beam stop and was omitted from the refinement. All H atoms positions were refined. Positions of carbon bound H atoms were freely refined, O bound H atoms were refined with an O—H distances restrained of 0.84 (2) Å. All *U*_iso_(H) values were refined.

### Crystal data

**Table:**

[Cd(C_9_H_7_O_2_)_2_(H_2_O)_2_]	*F*(000) = 444
*M**_r_* = 442.72	*D*_x_ = 1.705 Mg m^−^^3^
Monoclinic, *C*2	Mo *K*α radiation, λ = 0.71073 Å
*a* = 11.7872 (12) Å	Cell parameters from 4501 reflections
*b* = 5.3498 (5) Å	θ = 3.0–31.4°
*c* = 13.8817 (14) Å	µ = 1.30 mm^−^^1^
β = 99.913 (1)°	*T* = 100 K
*V* = 862.30 (15) Å^3^	Plate, colourless
*Z* = 2	0.28 × 0.09 × 0.02 mm

### Data collection

**Table:**

Bruker SMART APEX CCD diffractometer	2531 independent reflections
Radiation source: fine focus sealed tube	2529 reflections with *I* > 2σ(*I*)
Graphite monochromator	*R*_int_ = 0.021
ω and φ scans	θ_max_ = 31.4°, θ_min_ = 3.0°
Absorption correction: multi-scan (*SADABS*; Bruker, 2012)	*h* = −17→16
*T*_min_ = 0.617, *T*_max_ = 0.746	*k* = −7→7
5087 measured reflections	*l* = −20→19

### Refinement

**Table:**

Refinement on *F*^2^	Secondary atom site location: difference Fourier map
Least-squares matrix: full	Hydrogen site location: difference Fourier map
*R*[*F*^2^ > 2σ(*F*^2^)] = 0.020	All H-atom parameters refined
*wR*(*F*^2^) = 0.046	*w* = 1/[σ^2^(*F*_o_^2^) + (0.0239*P*)^2^] where *P* = (*F*_o_^2^ + 2*F*_c_^2^)/3
*S* = 1.05	(Δ/σ)_max_ = 0.002
2531 reflections	Δρ_max_ = 1.09 e Å^−^^3^
150 parameters	Δρ_min_ = −0.42 e Å^−^^3^
3 restraints	Absolute structure: Flack parameter determined using 1059 quotients [(*I*^+^)-(*I*^-^)]/[(*I*^+^)+(*I*^-^)] (Parsons *et al.*, 2013)
Primary atom site location: structure-invariant direct methods	Absolute structure parameter: 0.018 (14)

### Special details

**Table:**

Geometry. All e.s.d.'s (except the e.s.d. in the dihedral angle between two l.s. planes) are estimated using the full covariance matrix. The cell e.s.d.'s are taken into account individually in the estimation of e.s.d.'s in distances, angles and torsion angles; correlations between e.s.d.'s in cell parameters are only used when they are defined by crystal symmetry. An approximate (isotropic) treatment of cell e.s.d.'s is used for estimating e.s.d.'s involving l.s. planes.

### Fractional atomic coordinates and isotropic or equivalent isotropic displacement parameters (Å^2^)

**Table:**

	*x*	*y*	*z*	*U*_iso_*/*U*_eq_	
Cd1	1.0000	0.42839 (2)	1.0000	0.00869 (6)	
O3	0.90970 (17)	0.1439 (4)	1.07385 (17)	0.0152 (4)	
H3A	0.943 (3)	0.013 (5)	1.091 (3)	0.020 (10)*	
H3B	0.841 (2)	0.127 (10)	1.064 (4)	0.044 (14)*	
O1	0.96843 (16)	0.7270 (4)	0.87606 (14)	0.0116 (4)	
C1	0.8274 (2)	1.4604 (9)	0.65368 (19)	0.0130 (8)	
H1	0.910 (3)	1.43 (2)	0.661 (2)	0.027 (8)*	
O2	0.81721 (16)	0.6060 (4)	0.93838 (15)	0.0129 (4)	
C2	0.7702 (3)	1.6408 (5)	0.5914 (2)	0.0160 (5)	
H2	0.810 (3)	1.741 (8)	0.558 (3)	0.013 (9)*	
C3	0.6509 (3)	1.6611 (6)	0.5806 (2)	0.0170 (5)	
H3	0.615 (3)	1.787 (8)	0.540 (3)	0.020 (10)*	
C4	0.5903 (3)	1.5009 (6)	0.6322 (2)	0.0180 (6)	
H4	0.505 (3)	1.511 (7)	0.623 (3)	0.021 (10)*	
C5	0.6473 (3)	1.3212 (6)	0.6945 (2)	0.0163 (5)	
H5	0.605 (3)	1.209 (8)	0.726 (3)	0.020 (10)*	
C6	0.7674 (2)	1.2971 (5)	0.70604 (19)	0.0116 (5)	
C7	0.8322 (2)	1.1060 (5)	0.76880 (19)	0.0113 (5)	
H7	0.915 (3)	1.100 (7)	0.769 (3)	0.019 (10)*	
C8	0.7895 (2)	0.9423 (16)	0.82500 (18)	0.0134 (6)	
H8	0.708 (3)	0.92 (2)	0.831 (3)	0.031 (9)*	
C9	0.8621 (2)	0.7486 (5)	0.88228 (19)	0.0097 (4)	

### Atomic displacement parameters (Å^2^)

**Table:**

	*U*^11^	*U*^22^	*U*^33^	*U*^12^	*U*^13^	*U*^23^
Cd1	0.00734 (9)	0.00637 (10)	0.01212 (10)	0.000	0.00096 (6)	0.000
O3	0.0085 (9)	0.0093 (9)	0.0282 (11)	0.0011 (7)	0.0046 (8)	0.0044 (8)
O1	0.0089 (8)	0.0106 (9)	0.0152 (9)	0.0004 (7)	0.0022 (7)	0.0007 (7)
C1	0.0150 (10)	0.011 (2)	0.0127 (10)	−0.0026 (11)	0.0010 (8)	0.0008 (10)
O2	0.0082 (8)	0.0122 (9)	0.0179 (9)	−0.0003 (7)	0.0013 (7)	0.0047 (7)
C2	0.0233 (14)	0.0117 (12)	0.0128 (12)	−0.0010 (10)	0.0027 (10)	0.0027 (10)
C3	0.0237 (14)	0.0140 (13)	0.0122 (12)	0.0054 (11)	0.0001 (10)	0.0019 (10)
C4	0.0170 (12)	0.0210 (15)	0.0158 (13)	0.0058 (9)	0.0024 (11)	0.0034 (9)
C5	0.0158 (12)	0.0182 (13)	0.0152 (13)	0.0025 (10)	0.0038 (11)	0.0040 (10)
C6	0.0142 (12)	0.0113 (13)	0.0091 (11)	0.0020 (9)	0.0013 (9)	−0.0010 (9)
C7	0.0118 (11)	0.0110 (12)	0.0104 (11)	0.0014 (9)	−0.0002 (9)	0.0001 (9)
C8	0.0109 (9)	0.0130 (15)	0.0158 (9)	0.0069 (19)	0.0015 (7)	0.0020 (19)
C9	0.0100 (11)	0.0082 (11)	0.0103 (11)	−0.0015 (9)	−0.0003 (9)	−0.0012 (9)

### Geometric parameters (Å, º)

**Table:**

Cd1—O3^i^	2.208 (2)	O2—C9	1.269 (3)
Cd1—O3	2.208 (2)	C2—C3	1.392 (4)
Cd1—O1^i^	2.330 (2)	C2—H2	0.89 (4)
Cd1—O1	2.330 (2)	C3—C4	1.391 (4)
Cd1—O2	2.3753 (19)	C3—H3	0.94 (4)
Cd1—O2^i^	2.375 (2)	C4—C5	1.386 (4)
Cd1—C9	2.708 (3)	C4—H4	0.99 (4)
Cd1—C9^i^	2.708 (3)	C5—C6	1.403 (4)
O3—H3A	0.82 (2)	C5—H5	0.93 (4)
O3—H3B	0.80 (2)	C6—C7	1.469 (4)
O1—C9	1.276 (3)	C7—C8	1.328 (7)
C1—C2	1.390 (5)	C7—H7	0.98 (4)
C1—C6	1.403 (5)	C8—C9	1.485 (7)
C1—H1	0.98 (4)	C8—H8	0.99 (4)
			
O3^i^—Cd1—O3	92.86 (11)	C2—C1—C6	121.4 (3)
O3^i^—Cd1—O1^i^	141.89 (7)	C2—C1—H1	124 (5)
O3—Cd1—O1^i^	99.10 (8)	C6—C1—H1	114 (5)
O3^i^—Cd1—O1	99.10 (8)	C9—O2—Cd1	90.74 (15)
O3—Cd1—O1	141.89 (7)	C1—C2—C3	119.6 (3)
O1^i^—Cd1—O1	93.45 (10)	C1—C2—H2	120 (3)
O3^i^—Cd1—O2	126.04 (8)	C3—C2—H2	121 (2)
O3—Cd1—O2	87.88 (7)	C4—C3—C2	119.7 (3)
O1^i^—Cd1—O2	90.65 (7)	C4—C3—H3	122 (2)
O1—Cd1—O2	55.96 (7)	C2—C3—H3	118 (2)
O3^i^—Cd1—O2^i^	87.88 (7)	C5—C4—C3	120.8 (3)
O3—Cd1—O2^i^	126.04 (8)	C5—C4—H4	120 (2)
O1^i^—Cd1—O2^i^	55.96 (7)	C3—C4—H4	120 (2)
O1—Cd1—O2^i^	90.65 (7)	C4—C5—C6	120.4 (3)
O2—Cd1—O2^i^	132.85 (10)	C4—C5—H5	119 (3)
O3^i^—Cd1—C9	116.30 (8)	C6—C5—H5	120 (3)
O3—Cd1—C9	115.41 (8)	C5—C6—C1	118.2 (3)
O1^i^—Cd1—C9	90.85 (7)	C5—C6—C7	122.9 (3)
O1—Cd1—C9	28.08 (7)	C1—C6—C7	118.9 (3)
O2—Cd1—C9	27.95 (7)	C8—C7—C6	126.6 (3)
O2^i^—Cd1—C9	112.16 (8)	C8—C7—H7	117 (2)
O3^i^—Cd1—C9^i^	115.41 (8)	C6—C7—H7	116 (2)
O3—Cd1—C9^i^	116.30 (8)	C7—C8—C9	122.2 (2)
O1^i^—Cd1—C9^i^	28.08 (7)	C7—C8—H8	127 (5)
O1—Cd1—C9^i^	90.85 (8)	C9—C8—H8	111 (5)
O2—Cd1—C9^i^	112.16 (8)	O2—C9—O1	120.3 (2)
O2^i^—Cd1—C9^i^	27.96 (7)	O2—C9—C8	119.0 (3)
C9—Cd1—C9^i^	101.50 (11)	O1—C9—C8	120.6 (2)
Cd1—O3—H3A	119 (3)	O2—C9—Cd1	61.30 (13)
Cd1—O3—H3B	123 (4)	O1—C9—Cd1	59.28 (13)
H3A—O3—H3B	112 (5)	C8—C9—Cd1	174.6 (3)
C9—O1—Cd1	92.64 (15)		

Symmetry code: (i) −*x*+2, *y*, −*z*+2.

### Hydrogen-bond geometry (Å, º)

**Table:**

*D*—H···*A*	*D*—H	H···*A*	*D*···*A*	*D*—H···*A*
O3—H3*A*···O1^ii^	0.82 (2)	1.86 (2)	2.679 (3)	174 (4)
O3—H3*B*···O2^iii^	0.80 (2)	1.86 (3)	2.658 (3)	171 (5)

Symmetry codes: (ii) −*x*+2, *y*−1, −*z*+2; (iii) −*x*+3/2, *y*−1/2, −*z*+2.
